# Spatiotemporal evolution regional differences and decoupling effects of greenhouse gas emissions from animal husbandry in Henan Province

**DOI:** 10.1038/s41598-025-22285-8

**Published:** 2025-11-03

**Authors:** Yanyu Sha, Jiaqi Li, Hongbo Zhang, Chunbo Wei

**Affiliations:** 1 Department of Animal Science, College of Animal Science and Veterinary Medicine, Heilongjiang Bayi Agricultural University, Daqing, 163316 China; 2 Key Laboratory of Low-Carbon Green Agriculture in Northeastern China, Ministry of Agriculture and Rural Affairs, Beijing, 100125 China

**Keywords:** Animal husbandry, Greenhouse gas emissions, Space–time change, Regional differences, Tapio decoupling model, Markov chain, Climate sciences, Environmental sciences, Environmental social sciences

## Abstract

As global warming intensifies, the livestock industry has become one of the largest contributors to greenhouse gas (GHG) emissions, with its environmental impact increasingly drawing attention. Henan Province is a major contributor to these emissions, prompting this study to systematically investigate GHG emissions from the livestock industry in Henan Province. This study utilises panel data from 2001 to 2021 and employs the Life Cycle Assessment (LCA) method to estimate GHG emissions from the livestock industry, revealing their spatiotemporal changes and regional characteristics. Spatial analysis of GHG emissions from the livestock industry is conducted using the Theil index, the Tapio decoupling model, and Markov chains. The findings are as follows: (1) The total GHG emissions from the livestock industry exhibit a fluctuating downward trend, gradually forming a main GHG emission belt extending from the northwest to the southeast. (2) The Theil index shows an overall ‘declining trend,’ with inter-regional differences being the primary source of overall variation. (3) The overall decoupling state is primarily characterised by weak and strong decoupling, indicating that environmental protection and economic development in Henan Province are gradually moving toward coordination. (4) Regional GHG emission changes are constrained by their original emission types and reserves, exhibiting growth inertia and path dependence, with neighbouring types significantly influencing the transition of regional GHG emission types. Henan Province should formulate differentiated emission reduction policies and optimise the spatial layout of the livestock industry, which holds certain implications for other regions in achieving GHG emission reductions and livestock industry development.

## Introduction

Global temperature leapt up in the past two years, passing the + 1.5 °C level, and it will continue to rise for at least the next few decades^1^. The increase in GHG emissions is the main cause of global warming and urgently requires concerted international action to reduce emissions^2,3^. As the world’s largest carbon emitter, China has incorporated climate change into its national strategy and set ‘dual-carbon’ targets to promote the need for industries to actively fulfill the tasks of reducing emissions and increasing efficiency^4,5^. Within agriculture, animal husbandry has become the focus of attention due to its greater impact on the environment. Over recent years, animal husbandry has developed rapidly and has become a pillar industry of China’s agriculture and rural economy^6^. The 2023 China Statistical Yearbook indicates that the output value of animal husbandry in 2022 was 4,065.24 billion yuan, representing 26% of the total output value of agriculture, forestry, animal husbandry, and fishery, second only to agriculture, and higher than fishery and forestry. The growth of social economy promotes the increase of human demand for animal products^7^, which will not only expand the breeding scale and planting area of feed crops, but also produce toxic and harmful substances such as ammonia, sulfide and methane, causing serious pollution to the farm and its surrounding atmosphere, resulting in a large number of GHG emissions^8,9^. Therefore, it is very important to accelerate the low-carbon transformation of animal husbandry.

A significant number of scholars have conducted comprehensive research on the GHG emissions associated with animal husbandry from various perspectives, which provides new ideas for China to transition toward a more sustainable and environmentally conscious development model for animal husbandry. The methods of measuring animal husbandry GHG emissions in academia mainly include the Intergovernmental Panel on Climate Change (IPCC) coefficient method^10^, the Life Cycle Assessment (LCA)^11,12^, and the input–output method (I-O)^13,14^. Of these, the IPCC coefficient method and the LCA method are the most commonly employed^15^. The IPCC coefficient method mainly focuses on the carbon emissions in the production process of livestock and poultry farming, and it does not take into account the carbon emissions of the front-end and back-end of the livestock and poultry farming industry. In contrast, the LCA method covers the entire life cycle of a product, from the extraction of natural resources to the processing of raw materials, manufacturing, distribution, use of the product, and its eventual disposal or recycling, and therefore allows for a more specific and detailed assessment of the environmental footprint of a particular product or service^16–18^. Based on the above methods, the existing research has made a series of progress: Based on the LCA and IPCC coefficient method, He and Deng et al.^19^ analysed the spatial and temporal evolution of GHG emission intensity in China from 2000 to 2020. Zhao et al.^20^ used a spatial econometric model to analyse the impact of changes in the spatial structure of the livestock industry on methane emissions. Zhang et al.^21^ analysed the spatial and temporal patterns of carbon emissions from pig farming in 30 provinces (cities) in China and predicted the emissions from 2023 to 2032. The research conducted by Bai et al.^22^ examined the correlation between GHG emissions originating from animal husbandry and the trajectory of economic advancement within the Qinghai-Tibet Plateau. Du et al.^23^ quantified the carbon emissions of the livestock industry in 31 provinces in China from 2004 to 2020 using life cycle analysis. They also introduced a spatial transfer and share analysis method to assess the competitiveness and efficiency in carbon reduction of the industrial structure during the transition to low-carbon practices in the regional livestock sector. Jiang et al.^24^ studied the current status and future trends of decoupling CO_2_ emissions from the agricultural sector. Elahi et al.^25^ studied 165 agricultural green development zones to analyse changes in emissions, evaluate the decoupling between emission reductions and production value, and identify influencing factors. Liu et al.^26^ investigated the relationship between livestock GHG emissions and the decoupling of industrial development in 165 modern green agricultural development zones in China from 2010 to 2019. In addition, Luo et al.^27^ calculated family farms in Sichuan Province, showing that the carbon footprint of pork produced by large-scale farms was 4.29 kg CO_2_-eq, while the carbon footprint of free-range pork was 5.42 kg CO_2_-eq. Nejad et al.^28^ review explores the important impacts of methane emissions on livestock, especially cattle, and their significant impacts on climate change.

The existing research has laid a solid foundation for this paper to continue to carry out research in this field, but there are still some shortcomings: (1) Most studies focus on the national level or the macro regional level, and pay less attention to the systematic analysis of important emission reduction provinces (such as Henan Province and other major animal husbandry areas). (2) The IPCC emission factor method and LCA mainly focus on emission accounting, and the analysis of the dynamic evolution of emission paths (such as the inertia of type conversion) is insufficient. (3) Although existing studies have explored the relationship between emissions and the economy, the analysis of the causes of regional differences, spatial interaction effects (such as proximity effects), and the stage characteristics of the decoupling state is not sufficient.

In summary, this paper takes Henan Province, a major livestock-producing province in China, as its research subject and employs the Theil index, the Tapio decoupling model, and the Markov chain method to conduct a multi-dimensional analysis. The Theil index is used to analyse differences within and between regions, addressing the shortcomings of existing research in terms of analysing the causes of these differences; the Markov chain model reveals the dynamic transfer patterns of emission types and examines the spatiotemporal evolution trends; and the Tapio decoupling model is used to analyse the relationship between livestock industry GHG emissions and economic development. This study aims to clarify the spatiotemporal characteristics of GHG emissions from the livestock industry in Henan Province, the primary sources of regional differences, the dynamic evolution characteristics, and the degree of coordination with economic development, thereby providing a reference for formulating differentiated low-carbon development policies for the livestock industry.

Henan Province is a key production and processing base for livestock products in China, focusing on building a modern livestock production system, management system, and industrial system to promote high-quality development of the livestock industry and strongly support the construction of a modern agricultural powerhouse. While its economic strength continues to grow, it faces challenges such as worsening agricultural non-point source pollution and insufficient internal driving forces for green development. Although a series of emission reduction policies have been introduced to promote energy conservation, emission reduction, and pollution control in the livestock industry^29,30^, there is a lack of systematic understanding of emission patterns. Therefore, this study not only fills the gap in multi-dimensional analysis of GHG emissions from livestock farming at the provincial level, but also provides valuable reference for other regions in achieving GHG emission reductions from livestock farming and promoting the development of the livestock industry.

## Materials and methods

### Overview of the study area

**Fig. 1:**
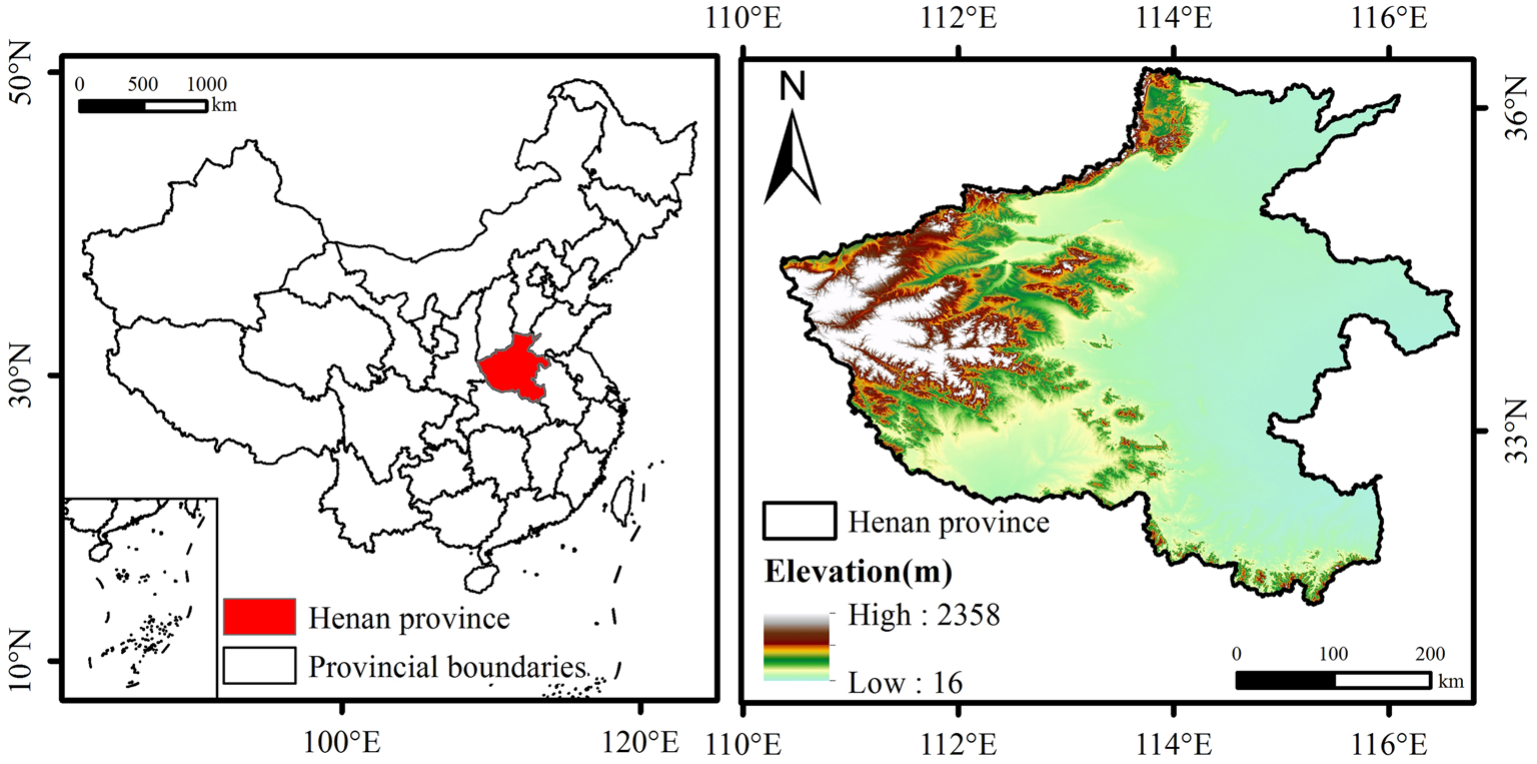
Geographic location of the study area. This was created using ArcGIS software to create a map of the geographical location of the study area. The software access link is https://cloudcenter.tianditu.gov.cn/administrativeDivision. The map review number is GS (2024) 0650.

Henan Province is located on the middle and lower reaches of the Yellow River in east-central China (Fig. 1), bounded by latitude 31°23′–36°22′N and longitude 110°21–116°39′E, bordered by Shandong and Anhui in the east, Hebei and Shanxi in the north, Shaanxi in the west and Hubei in the south, and presenting a situation of looking north to south and bearing east to west, which makes Henan Province an important transport hub and a large agricultural province. The total area of the province is about 167,000 square kilometres, and the terrain is dominated by plains, which account for 1.73% of the country’s total land area, and mountains and hills, which account for 26.6% and 17.7% respectively^31^. Most of Henan is located in the warm temperate zone, the south crosses the subtropics, belongs to the continental monsoon climate of the transition from the northern subtropical to the warm temperate zone, and also has the characteristics of the transition from the plain to the hilly and mountainous climate from east to west, which is suitable for growing a variety of crops and pig breeding.

Henan Province is a major grain-producing region and also a significant producer of livestock products. In recent years, the scale of livestock production has expanded significantly, with the five key industries—pork, beef cattle, dairy cattle, poultry, and sheep—growing rapidly. Henan ranks among the top provinces in China in terms of meat, poultry, eggs, and dairy production. As one of China’s largest pig-raising provinces, Henan had a pig inventory of 40.31 million head and a breeding sow inventory of 3.73 million head in 2024, both ranking first nationwide. Leveraging leading enterprises such as Mu Yuan and Shuanghui, the province has established a complete industrial chain covering breeding, farming, slaughtering, and processing. Currently, the comprehensive scale rate of livestock and poultry farming in the province has reached 73.3%, with 80% for pigs, 73% for egg-laying chickens, and 81% for meat chickens^32^. Some livestock breeds have already achieved modernisation. Additionally, Henan Province possesses abundant local superior breed resources, including provincial-level protected livestock and poultry breeds such as the Nanyang cattle, Jia County red cattle, and Huai pigs. In the field of manure management, mature and stable treatment and utilisation models have been established, such as storage plus agricultural utilisation, anaerobic digestion plus agricultural utilisation, and energy-ecological models. Resource utilisation levels have significantly improved, and the regulatory framework is gradually being perfected. Despite significant progress in scale and technology, Henan Province’s livestock industry still faces severe challenges in achieving carbon emission reduction targets^33^.

Based on previous literature, Henan Province has been divided into five regions—East Henan, Central Henan, South Henan, West Henan, and North Henan—according to the topography, landforms, and geographical characteristics of each city^34^. The cities belonging to each region are listed in Table 1. The central part of the Huanghuaihai Plain in East Henan is the main grain-producing area, with abundant feed resources, and is primarily engaged in pig, cattle, and poultry farming. Central Henan, as the provincial economic and transportation hub, has developed highly concentrated industries such as slaughtering and processing, dairy products, and feed production. Southern Henan is the largest livestock farming region in the province, with significant advantages in pig farming, waterfowl, and beef cattle farming. Henan West is predominantly mountainous and hilly terrain, with a well-developed grass-fed livestock industry and a variety of local specialty breeds. Henan North has favourable natural and agricultural conditions, serving as a major grain and cotton production area in China, particularly a key base for high-quality wheat production, while also functioning as an important base for livestock production and processing in Henan Province.

**Table 1:** Regional division of Henan Province.

Serial number	Geographic division	City
1	Eastern	Kaifeng, Shangqiu, Zhoukou
2	Central	Zhengzhou, Pingdingshan, Xuchang, Luohe
3	Southern	Nanyang, Xinyang, Zhumadian
4	West	Luoyang, Jiaozuo, Sanmenxia, Jiyuan
5	North	Anyang, Hebi, Xinxiang, Puyang

### Estimation of GHG emissions from livestock farming

**Fig. 2:**
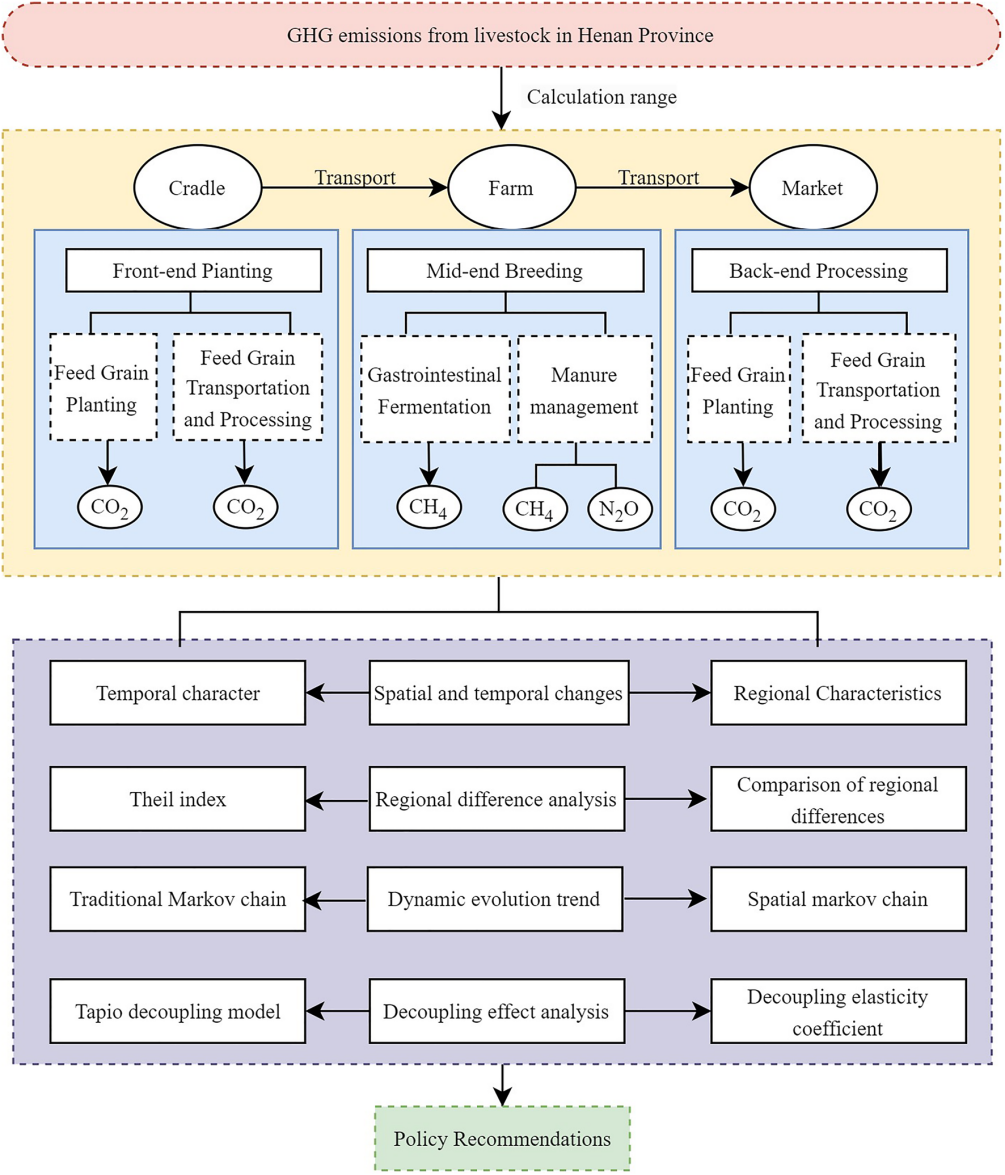
GHG emission estimation and analysis framework of the livestock farming production system.

This study focuses on GHG emissions caused by livestock farming, mainly including Carbon dioxide (CO_2_), Methane (CH_4_), and Nitrous Oxide (N_2_O)^35^. According to the LCA method, GHG emissions from livestock farming are divided into six links: gastrointestinal fermentation of livestock and poultry, manure management system, energy consumption of livestock and poultry breeding, feed grain planting, feed grain transportation and processing, and slaughter of livestock and poultry products^36^. All GHG emissions are converted to CO_2_-eq based on global warming potential (GWP) to facilitate the calculation of GHG emissions. The estimation and analysis framework of GHG emissions from the livestock farming production system in Henan Province is shown in Fig. 2^33^.

The upper half of Fig. 2 comprises three stages: the cradle stage refers to upstream farming, encompassing feed production (such as corn and soybean meal cultivation), livestock breeding, and inputs of energy and agricultural inputs (fertilisers, pesticides); the farm stage refers to midstream farming, including animal intestinal fermentation (CH_4_), manure management systems (CH_4_ and N_2_O), and energy consumption at livestock farms (such as heating and ventilation); the market stage refers to downstream processing, covering the downstream processes from farm to consumer, including livestock slaughtering, processing (such as refrigeration and packaging), transportation, and retail processes. The lower half represents spatial analysis.

### Formula for estimating GHG emissions from livestock farming

*Feed grain cultivation.* A feed can be divided into roughage and concentrate. Livestock and poultry are mainly based on feed raw materials such as soybeans, corn, and wheat. Roughage is a by-product of primary processing, and the GHG produced during the production process is negligible^37^. In this part, only GHG emissions caused by concentrated feed planting are calculated. The calculation formula for CO_2_ emission from feed grain planting is as follows:

1 $$\text{EFC} = \sum_{i=1}^{n}{Q}_{T}\cdot t\cdot {q}_{i}\cdot {ef}_{j1}$$

*Feed grain transportation and processing.* The processing of feedstuffs, such as corn, soybeans, wheat, and so forth, entails a series of operations, including transportation, ingredients, and granulation during the production process to become livestock and poultry feed. The GHG emissions generated here should also be included in the boundaries of the livestock farming system^33^. The calculation formula is as follows:

2 $$\text{EFT} = \sum_{i=1}^{n}{Q}_{T}\cdot t\cdot {q}_{i}\cdot {ef}_{j2}$$

In the formula, EFC is the CO_2_ emissions generated by the feed grain planting link. EFT signifies the CO_2_ emissions generated as a consequence of the processing and transportation of feed grain. Q_T_ is the annual output of T-type livestock and poultry products. The consumption factor per unit of livestock and poultry products, t, is a variable that can be calculated. The proportion of grain in the formulation of livestock and poultry feeds in category i is represented by q_i_. The CO_2_ emission factor for grain in category j, ef_j1_, and the CO_2_ emission factor for processing and transporting grain in category j, ef_j2_, are presented in Table 2^33^.

**Table 2:** The GHG emission coefficient of each link of livestock farming.

Link	Symbol	Emission coefficient	Values	Unit	Reference Source
Feed grain cultivation	ef_j1_	CO_2_-eq emission Factor of corn	1.50	t/t	^37^
		CO_2_-eq emission Factor of wheat	1.22	t/t	
Feed grain transport &processing	ef_j2_	CO_2_-eq emission Factor of corn	0.0102	t/t	^36^
		CO_2_-eq emission Factor of soybean	0.1013	t/t	
		CO_2_-eq emission Factor of wheat	0.0319	t/t	
Livestock feeding	ef_e_	CO_2_ emission factor of electricity consumption	0.9734	t/(MW·h)	
	price_e_	Breeding electricity unit price	0.4275	CNY/(KW·h)	
	ef_c_	Coal combustion CO_2_ emission factor	1.98	t/t	
	price_c_	Unit price of coal	800	CNY /t	
Livestock products processing	MJ_u_	Energy consumption for processing pork products	3.76	MJ/kg	^38^
		Energy consumption for processing beef products	4.37	MJ/kg	
		Energy consumption in the processing of mutton products	10.4	MJ/kg	
		Energy consumption in the processing of poultry meat products	2.59	MJ/kg	
		Energy consumption in the processing of milk products	1.12	MJ/kg	
		Energy consumption in the processing of poultry and egg products	8.16	MJ/kg	
	e	Electric heating value	3.60	MJ/(KW·h)	–
Other conversion coefficients	${\text{GWP}}_{{\text{CH}}_{4}}$	CH_4_ Global warming potential	27	–	^21^
	${\text{GWP}}_{{\text{N}}_{2}{\text{O}}}$	N_2_O Global warming potential	273		

*Gastrointestinal fermentation.* The gastrointestinal tract of livestock functions under anaerobic conditions, and rumen fermentation in ruminants produces a substantial quantity of CH_4_ gas, representing a significant source of CH_4_ emissions^39,40^. The following formula is used to calculate the emissions of CH_4_ from the gastrointestinal fermentation of livestock and poultry:

3 $$\text{EFG} = \sum_{\text{i=1}}^{\text{n}}{{\text{APP}}}_{\text{i}} \cdot {\text{ef}}_{\text{i1}}$$

In the formula, EFG is the CH_4_ emissions of livestock and poultry gastrointestinal fermentation; The variable i denotes the livestock and poultry rearing category; The term APP_i_ is the average annual feeding amount of i-type livestock and poultry; The symbol ef_i1_ represents the CH_4_ emission coefficient of gastrointestinal fermentation of class i livestock and poultry (Table 3)^33^.

**Table 3:** GHG emission factors of livestock and poultry gastrointestinal fermentation and manure management systems.

Species	CH_4_[kg/(head. a)]		N_2_O (kg/head/a)	Reference source
	Gastrointestinal fermentation	Manure management		
Cattle	52.90	3.31	0.85	^41^
Pig	1.00	3.50	0.53	^42^
Sheep	5.00	0.16	0.33	
Mule	10.00	0.90	1.39	
Donkey	10.00	0.90	1.39	
Horse	18.00	1.64	1.39	
Rabbit	0.254	0.08	0.02	^43^
Poultry	–	0.02	0.02	

*Manure management system.* After anaerobic decomposition of livestock and poultry manure, a large amount of CH_4_ gas is released. At the same time, the nitrogen contained in the manure can also undergo nitrification and denitrification under suitable conditions, which in turn leads to N_2_O emissions^44,45^. The estimation of GHG emissions from manure management systems involves a two-step process.

The formula used to calculate methane emissions in the context of manure management is as follows:

4 $$\text{EFM} = \sum_{\text{i=1}}^{\text{n}}{{\text{APP}}}_{\text{i}}\cdot{\text{ef}}_{\text{i2}}$$

The following formulae are used to calculate N_2_O emissions from manure management systems:

5 $$\text{EFS} = \sum_{\text{i=1}}^{\text{n}}{{\text{APP}}}_{\text{i}}\cdot{\text{ef}}_{\text{i3}}$$

In the formula, EFM and EFS refer to the emissions of CH_4_ and N_2_O, respectively, from livestock and poultry manure management systems; livestock feeding categories are denoted by i; APP_i_ represents the average annual feeding of livestock in category i; the ef_i2_ and ef_i3_ are the CH_4_ and N_2_O emission factors of the i-type livestock and poultry manure management system, as shown in Table 3^33^.

*Energy consumption.* Livestock and poultry in the breeding process will consume significant quantities of energy, including electricity, coal, and other energy, which will directly or indirectly cause carbon emissions He and Lin et al.^38^. Accordingly, the CO_2_ emissions generated by livestock and poultry rearing can be calculated using the following formula:

6 $$\text{EFE} = \sum_{\text{i=1}}^{\text{n}}{{\text{NAPA}}}_{\text{i}}\cdot\frac{{\text{cost}}_{\text{ie}}}{{\text{price}}_{\text{e}}}\cdot{\text{ef}}_{\text{e}}\text{+}\sum_{\text{i=1}}^{\text{n}}{{\text{NAPA}}}_{\text{i}}\cdot\frac{{\text{cost}}_{\text{ic}}}{{\text{price}}_{\text{c}}}\cdot{\text{ef}}_{\text{c}}$$

In the formula, EFE represents the carbon dioxide emissions generated by energy consumption in the process of livestock and poultry feeding; livestock feeding categories are denoted by i; NAPA_i_ is the average annual feed of category i livestock and poultry; cost_ie_ is the electricity expenditure per head (only) of Class i livestock and poultry; the price_e_ is used in this context to refer to the unit price of electricity used for livestock and poultry breeding; ef_e_ represents the CO_2_ emission factor for electrical energy consumption (Table 2); cost_ic_ is the coal expenditure per head (only) of Class i livestock and poultry; price_c_ denotes the unit price of coal utilized for livestock and poultry breeding; ef_c_ represents the CO_2_ emission factor for coal burning (Table 2)^33^.

*Product processing.* After the live livestock and poultry are transported to the slaughterhouse, they must be disinfected, packaged, and processed to make them a commodity for sale in the market, and at the same time, they will cause certain GHG emissions^33^. The calculation formula is as follows:

7 $$\text{EFP} = \sum_{\text{i=1}}^{\text{n}}{{\text{Q}}}_{\text{T}}\cdot\frac{{\text{MJ}}_{\text{u}}}{{\text{e}}_{\text{n}}}{\text{ef}}_{\text{e}}$$

In the formula, EFP refers to CO_2_ emissions produced by the slaughtering and processing of livestock and poultry; Q_T_ is the annual production of livestock and poultry products in category T; MJ_u_ is the energy consumption per unit of livestock slaughtering and processing in category u. e_n_ denotes the calorific value of one unit of electricity, while ef_e_ signifies the CO_2_ emission factor for electrical energy consumption (Table 2)^33^.

Following the LCA method, the total GHG emission calculation formula for livestock farming is as follows:

8 $$ETotal = EFC + EFT + \left( {EFG \cdot GWP_{{CH_{4} }} + EFM \cdot GWP_{{CH_{4} }} + EFS \cdot GWP_{{N_{2} O}} } \right) + EFE + EFP$$

In the formula, the term ETotal represents the total GHG emissions from livestock; EFC, EFT, EFG, EFE, and EFP refer to the emissions from feed grain cultivation, feed grain processing, and transport, gastrointestinal fermentation, feeding energy consumption, and product processing, respectively. EFM and EFS represent the emissions of CH_4_ and N_2_O from manure management systems; ${\text{GWP}}_{{\text{CH}}_{4}}$ and ${\text{GWP}}_{{\text{N}}_{2}{\text{O}}}$ denote the global warming potentials of CH_4_ and N_2_O respectively.

### Theil index

The Theil index is a method for analysing regional differences and spatial decomposition. It can be used to analyse overall regional differences, inter-regional differences, and changes in regional differences, as well as to analyse the causes of the differences. This paper investigates the spatial disparity in GHG emissions from livestock farming in Henan Province using the Theil index, as proposed by Theil and Uribe et al.^46^ and Shi et al.^47^. The calculation of the Theil index is as follows:

9 $$T=\frac{1}{n} \cdot \sum \limits_{i=1}^{n}\frac{{E}_{i}}{\mu }ln\frac{{E}_{i}}{\mu }$$

Among them, n is the number of observation samples, that is, 18 cities in Henan Province; μ is the average value of GHG emissions from livestock farming in 18 cities of Henan Province; The E_i_ variable represents the GHG emissions from livestock farming in city i. The numerical interval of the Theil index is [0, 1], whereby a smaller value indicates a smaller regional difference. Conversely, a higher value indicates a more significant regional difference^33,48,49^.

### Tapio decoupling model

The decoupling theory was first proposed by the Organisation for Economic Co-operation and Development (OECD) in "Indicators for Measuring the Decoupling of Economic Growth and Environmental Impacts," published in 2002, which explored the relationship between environmental pollution and the economy^38^. Petri Tapio, by introducing the elasticity method, classified the decoupling states into eight states, such as strong decoupling and weak decoupling, and established the "Tapio decoupling model"^50^. Carbon decoupling is an idealised process in which the relationship between economic growth and GHG emissions continuously weakens or even disappears, i.e., energy consumption is gradually reduced while economic growth is achieved^51^. This paper further investigates the decoupling relationship between GHG emissions and economic growth in the livestock sector in Henan Province. Based on the different value ranges of the decoupling elasticity coefficient, the carbon decoupling of the livestock industry can be classified into eight types^52,53^, which are specifically categorised as shown in Table 4. The formula is as follows:

**Table 4:** Tapio decoupling elastic model judgment criteria^52^.

Type of decoupling	Decoupling states	∆CO_2_	∆GDP	Decoupling elasticity values (E)
Decoupling	Strong decoupling	< 0	> 0	E < 0
	Weak decoupling	> 0	> 0	0 ≤ E < 0.8
	Recessive decoupling	< 0	< 0	E > 1.2
Negative decoupling	Weak negative decoupling	< 0	< 0	0 ≤ E < 0.8
	Strong negative decoupling	> 0	< 0	E < 0
	Expansive negative decoupling	> 0	> 0	E > 1.2
Coupling	Expansive coupling	> 0	> 0	0.8 ≤ E ≤ 1.2
	Recessive coupling	< 0	< 0	0.8 ≤ E ≤ 1.2

10 $$D_{(C,G)}=\frac{ \Delta \text{C/C}}{ \Delta \text{G/G}}$$

Among them, D_(C, G)_ represent the decoupling index of carbon emissions and GDP growth; C denotes the carbon emissions of the base year; G is the gross output value of livestock in Henan Province for the base year; ΔC is the difference between the carbon emissions of the current year and the base year; ΔG is the difference between the GDP of the current year and the base year. The criteria for determining the decoupling elasticity model are shown in Table 4.

### Markov chain

The concept of Markov chains was proposed by Andrey Markov and refers to a probabilistic process of discrete events in statistics that exhibit Markovian properties. In this probabilistic process, if the current state information remains unchanged, the transition probability from the current state to the next state does not depend on the previous state. Each state change is called a transition, and the probability of transitioning from one state to another is called the transition probability. The matrix composed of all transition probabilities is called the transition probability matrix^54^. Randomness, stability, and the lack of an aftereffect are advantages of the Markov chain. It is the best way to investigate the transfer probability, state space–time transfer path, and evolution law of the regional development level.

GHG emissions from livestock farming in Henan Province have undergone self-adjustment or been affected by surrounding factors, causing changes in GHG emissions over a certain period. This paper uses Markov chain analysis to study the probability of GHG emissions in a given unit shifting from one type of value to another. Using traditional Markov chains, this study examines the probability distribution of GHG emission levels at high or low levels and the stable transfer within a certain region, describing the dynamic evolution characteristics of GHG emission levels in Henan Province’s livestock industry^55^. This method combines all annual sample data and categorises them into K types based on certain standards. The state transition probability p_ij_ of the sample value of the i-th type (i = 1, 2, …, k) in year t transforming into the j-th type (j = 1, 2, …, k) in year t + T forms a k-order square matrix, i.e., the traditional Markov transition probability matrix^56,57^.

Traditional Markov chains do not consider the influence of neighbouring regions on the transition of state types in a given region. Changes in regional GHG emissions are not only influenced by the region’s proactive adjustments but also by incentive measures in surrounding regions^58^. Therefore, by incorporating spatial effects into the matrix, a spatial Markov chain can be obtained. Spatial Markov chains are used to determine whether the GHG emission levels of ‘neighbouring’ regions influence the transition of GHG emissions in the region. This is achieved through comparative analysis of Markov transition matrices under different spatial lag types^59^.

For a set of random variables in a probability space, at time t^60^:

11 $${\text{p}}\left\{{\text{X}}_{\text{n}}\text{+1=}{\text{x}}_{\text{in}}\text{+1|}{\text{X}}_{1}\text{=}{\text{x}}_{\text{i1}}\text{,}{\text{X}}_{2}\text{=}{\text{x}}_{\text{i2}}, \ldots {\text{X}}_{\text{n}}\text{=}{\text{x}}_{\text{in}}\right\}\text{=}{\text{p}}\left\{{\text{X}}_{\text{n}}\text{+1=}{\text{x}}_{\text{in}}\text{+1|}{\text{X}}_{\text{n}}\text{=}{\text{x}}_{\text{in}}\right\}$$

The probability p_ij_ of transitioning from state xi to state x_j_ is called the state transition probability. The state transition probability matrix is calculated using the maximum likelihood estimation method, and the Markov distribution after state transition is obtained through this matrix, as shown in the formula:

12 $$\widehat{{\text{p}}_{\text{ij}}}\text{=}{\text{n}}_{\text{ij}}\text{/}{\text{n}}_{\text{i}}$$

In this context, n_ij_ denotes the number of transitions from state i to state j, and ni denotes the total number of occurrences of state i. In a spatial Markov chain, a spatial weight matrix is introduced and divided into K groups in the same manner, resulting in a transition probability matrix of size (K × K) × K.

### Data source

The data used in this paper on cattle, pigs, sheep, poultry, milk, pork, beef, mutton, poultry meat, poultry eggs, etc. in Henan Province and each prefecture-level city are obtained from the Henan Statistical Yearbook, Henan Survey Yearbook, Henan Rural Statistical Yearbook and each prefecture-level city statistical yearbook of the past years (2002–2022), and the weighted average method is used to fill in some of the missing values. Data on the amount of grain used by each type of livestock and poultry, the output of staple foods, and the cost of electricity and coal are obtained from the National Compendium of Costs and Benefits of Agricultural Products over the years.

## Results

### Analysis of spatial and temporal changes in GHG emissions from livestock farming in Henan Province

#### Temporal character analysis

**Fig. 3:**
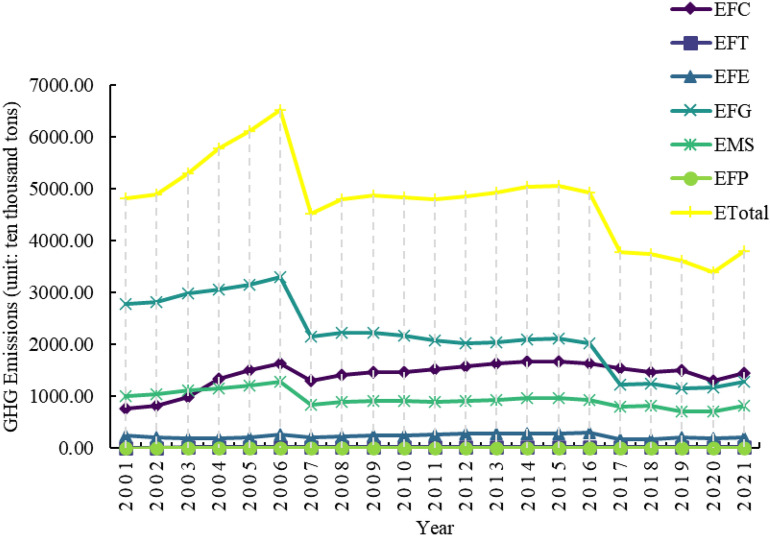
GHG emissions from livestock farming and various segments in Henan Province.

As shown in Fig. 3, GHG emissions from livestock farming in Henan Province decreased significantly from 4829.90 ten thousand tons in 2001 to 3805.48 ten thousand tons in 2021, with an average annual decrease rate of 1.18%^33^. However, there were two more significant decreases in livestock GHG emissions in Henan Province during this period. The details are as follows:

1. From 2001 to 2007, GHG emissions from livestock farming in Henan Province decreased from 4829.90 to 4533.08 ten thousand tons. From this stage, together with a series of pro-agriculture policies^33,61^, especially the implementation of the grain subsidy policy, the area under major crops in Henan Province has continued to expand, and the consumption of agricultural materials has increased. However, it is still dominated by the crude agricultural production model, which uses a large number of inputs such as pesticides, chemical fertilisers, and agricultural films in the process of increasing production, with insufficient consideration for the ecological environment^33,62^. In addition, the level of agricultural technology is backwards, making it difficult to keep up with the expansion of the scale of agriculture. There are shortcomings such as poor quality and palatability of feed processing, not easy to digest, etc., and the belching and exhausting of livestock lead to a large number of GHG emissions. In 2007, the livestock industry was hit by outbreaks of swine blue ear disease and avian influenza, which impacted production capacity and restricted the production cycle of livestock products. These unfavourable factors, combined with the inability of the market to quickly recover supply in the short term, led to a sharp decline in GHG emissions from the livestock industry. In addition, between 2001 and 2007, Henan Province’s livestock production’s GHG emissions decreased by 3.19% and 4.21%, respectively, due to energy consumption in feeding and manure management (Table 5).

**Table 5:** Rates of change of GHG emissions and their components from livestock farming in Henan Province, 2001–2007 and 2015–2021.

Emissions from different sectors	2001–2007 year	2015–2021 year
ETotal	− 1.05%	− 4.67%
EFC	9.08%	− 2.41%
EFT	9.50%	− 2.82%
EFE	− 3.19%	− 4.42%
EFG	− 4.21%	− 7.96%
EMS	− 2.79%	− 2.49%
EFP	1.83%	− 0.27%
2. During the period 2015–2021, GHG emissions from the livestock sector in 2021 showed a slight recovery, with fluctuations, but a more pronounced overall downward trend. It decreased from 5069.75 ten thousand tons in 2015 to 3805.48 ten thousand tons in 2021. Through industrial restructuring, Henan Province reduced its cattle inventory by 5.337 million head (a decrease of 57.1%) during this period, with the 2021 inventory amounting to only 42.9% of the 2015 level. This data indicates that the reduction in cattle inventory has made a significant contribution to GHG emission reductions in Henan Province’s livestock sector. Although Henan Province has aggressively developed large-scale livestock farming and actively responded to the national policy of developing low-carbon animal husbandry in recent years, some farmers have withdrawn from animal husbandry due to the lengthy production cycle and high production costs, which has caused the province’s cattle population to decline annually. To fully strengthen agricultural pollution control and ecological economic development, the "14th Five-Year Plan for Ecological Environmental Protection and Ecological Economic Development of Henan Province" explicitly outlines the following goals: enhancing the designation of no-farming zones and management systems; strengthening the idea of a "combination of planting and rearing"; encouraging the creative use of livestock and poultry manure throughout the county; and concentrating on agricultural counties and large-scale farms as key areas. Henan Province has made significant progress in lowering GHG emissions from livestock farming through the implementation of several national policies on green development and low-carbon animal husbandry^63–65^. The reductions in emissions resulting from gastrointestinal fermentation and feeding energy consumption are 7.96% and 4.42%, respectively (Table 5).
  Additionally, as shown in Fig. 4, the correlation coefficient and significance between GHG emissions and each livestock farming link in Henan Province were computed using the Pearson correlation analysis method. During the period 2001–2021, ETotal showed significance with EFG and EMS, with correlation coefficients of 0.924 and 0.946, respectively. It means that there is a positive correlation between the total amount of emissions and EFG and EMS. The decrease in GHG emissions caused by EFG and EMS is an important reason for the observed shift in GHG emissions from livestock farming in Henan Province during this period. A significant correlation was observed between the ETotal and those caused by EFC, EFG, EMS, and EEP from 2001 to 2007. The correlation coefficients were 0.758, 0.758, 0.954, and 0.927, respectively. This means that there is a positive correlation between total emissions and several factors, including EFC, EFG, EMS, and EFP. It reflects that the reduction of GHG emissions caused by EFC, EFG, EMS, and EFP is an important reason for the change in GHG emissions from livestock farming in Henan Province during this period. From 2015 to 2021, the GHG emissions of livestock farming in Henan Province were all significant, with values of 0.881, 0.937, 0.914, 0.990, 0.927, and 0.870. Furthermore, the correlation coefficient values were all greater than 0, indicating a positive correlation between the GHG emissions of livestock farming in Henan Province and each link. This suggests that, in addition to direct emissions, indirect emissions are also a significant contributing factor to the observed decrease in GHG emissions from livestock farming in Henan Province during this period^33^.

**Fig. 4:**
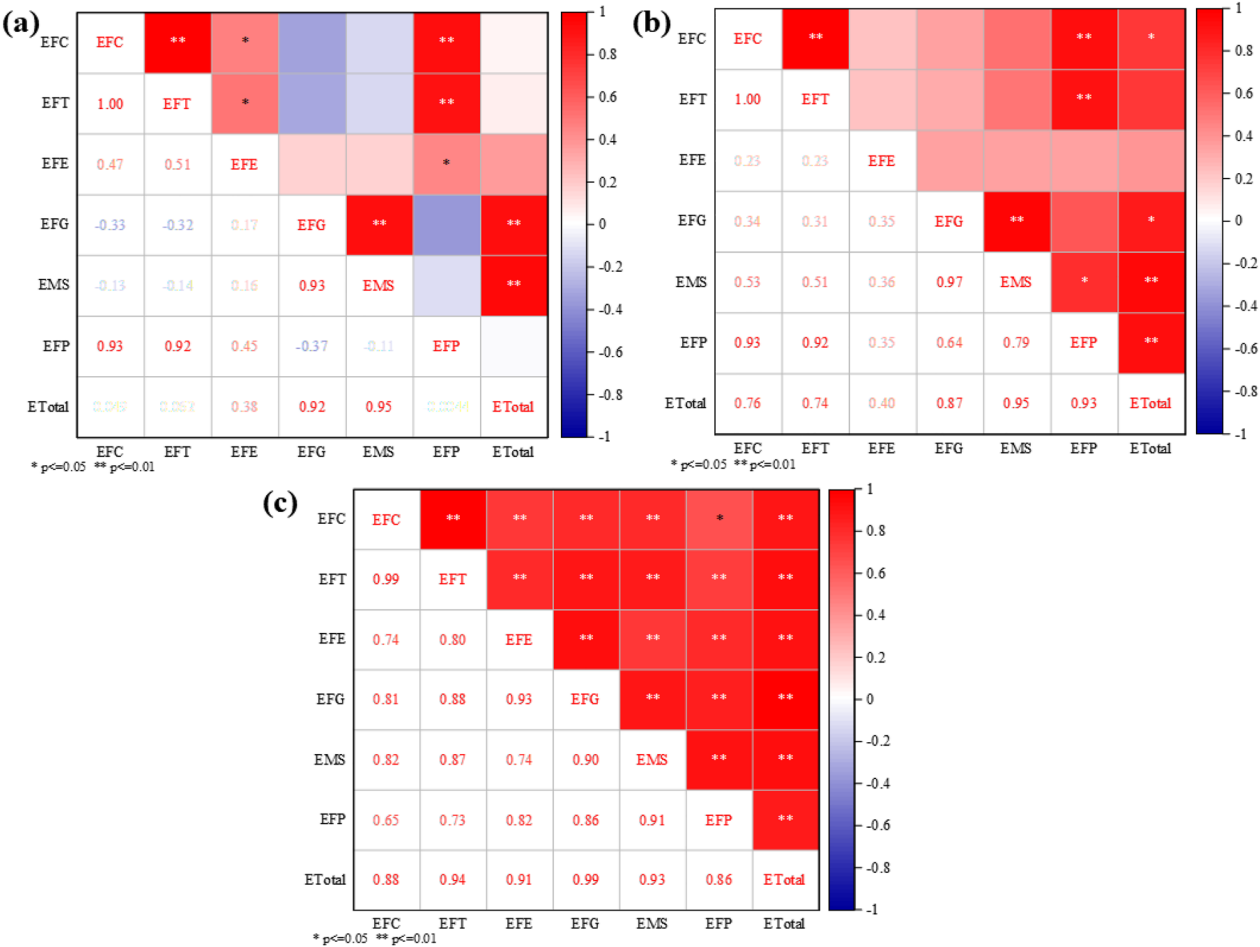
Results of correlation analysis between GHG emissions from livestock farming and various segments in Henan Province, (**a**) 2001–2021, (**b**) 2001–2007, and (**c**) 2015–2021.

#### Regional characteristics analysis

**Fig. 5:**
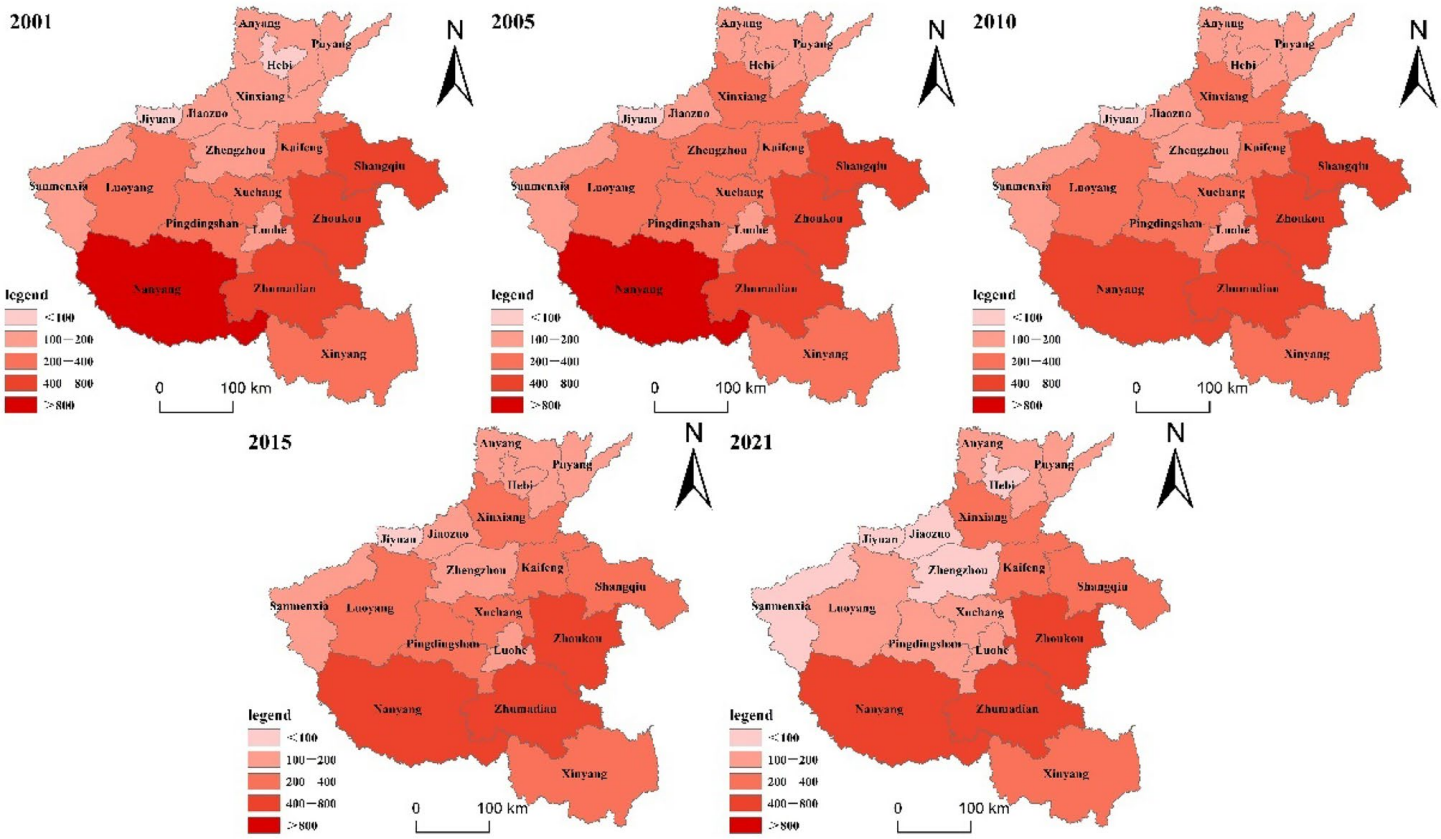
GHG emissions from livestock farming in Henan Province by prefecture-level cities in some years (unit: ten thousand tons). This was created using ArcGIS software to map China’s Henan Province. The software access link is https://cloudcenter.tianditu.gov.cn/administrativeDivision. The map review number is GS (2024) 0650.

Livestock GHG emissions are divided into five types: I, I, I, IV, and V, which correspond to the light, medium, heavy, and super-heavy GHG emissions of livestock farming (Fig. 5). During the last 20 years, the overall fluctuation of GHG emissions from livestock farming in Henan Province has decreased, and the spatial pattern has changed significantly, with the number of low areas increasing by 3 (Sanmenxia, Jiaozuo, Zhengzhou), the number of light areas decreasing by 3 (Luoyang, Pingdingshan, Xuchang), the number of medium areas increasing by 1 (Xinxiang), and the number of heavy areas decreasing by 1 (Shangqiu). The difference between Jiyuan, which emitted the least, and Nanyang, which emitted the most in 2001, was 32 times in terms of regional GHG emissions extremes. In 2021, the difference between Zhumadian, which emitted the most, and Jiyuan, which emitted the least, was 19 times. It is evident that during the past 20 years, there has been a decline in the disparity in total GHG emissions from livestock production across different regions.

From 2001 to 2021, Henan Province will progressively develop a significant GHG emission zone that stretches from northwest to southeast, as shown in Fig. 5. The demand for livestock and poultry products nationwide is still rising as living standards rise. Due to the different regional development patterns, the pressure of livestock production is naturally concentrated in the dominant areas. In 2001, GHG emissions from livestock farming accounted for 36.55% of the province’s total emissions in areas with a high level of economic development (Zhengzhou, Nanyang, Luoyang, Zhoukou, etc.). In 2021, their share will decrease to 30.52%. From 2001 to 2021, the GHG emissions of livestock farming in Zhumadian, Nanyang, and Zhoukou were above the heavy level, ranking among the top 3. As the main development area of livestock farming in Nanyang, the proportion of GHG emissions from livestock farming in the total GHG emissions from livestock farming in the province decreased from 17.40 to 13.02%, from super heavy to heavy. Nanyang, Xinyang, Zhumadian, and Zhoukou, which are major livestock farming cities in Henan Province, accounted for 48.15% of the total GHG emissions from livestock farming in the province, down from 48.15 to 44.14%^33^.

### Trend analysis of GHG emission changes in different regions of Henan Province’s livestock farming industry

Different cities in Henan Province have different GHG emissions from livestock farming, and the Theil index can be used to determine the GHG emissions gap under various levels of economic development. Additionally, the relationship between GHG emissions and economic growth in various regions can be examined. The trends and regional variations in GHG emissions across Henan Province’s various regions are displayed in Table 6. Overall, the GHG emissions and economic level of cities in Henan Province showed a decreasing and increasing trend, but the relative change trend of regional disparities was different. Since 2006, the Theil index of GHG emissions in Henan Province has shown a downward trend year by year, and the Theil index has decreased from 0.1064 to 0.0978 year by year, and the regional disparity in emissions between regions has gradually narrowed. Compared with the proportion of intra-regional and inter-regional contributions, the contribution of GHG emissions in Henan Province accounted for 22–33%, which showed a downward trend year by year. On the contrary, the interregional contribution of GHG emissions in Henan Province accounted for 66–77%, but it showed an increasing trend year by year. In contrast, the Theil index of economic development in Henan Province showed an upward trend, decreasing and increasing, from 0.0777 to 0.0581 in 2011, and then slowly increasing to 0.0827. If we further decompose the Theil index of economic development into intra-regional and inter-regional contributions, we can see that the intra-regional contribution is stable at 19–32%, and the inter-regional contribution is stable at 68–80%^33^

**Table 6:** Comparison of regional differences and contributions to GHG emissions and economic development in Henan’s livestock farming industry, 2001–2021.

Year	GHG Emissions			Economic development		
	Thiel index	Intra-regional contribution/%	Inter-regional contribution/%	Thiel index	Intra-regional contribution/%	Inter-regional contribution/%
2001	0.1248	25.57	74.43	0.0777	25.25	74.75
2002	0.1208	27.17	72.83	0.0754	27.22	72.78
2003	0.1152	28.03	71.97	0.0844	26.02	73.98
2004	0.1097	27.84	72.16	0.0730	25.14	74.86
2005	0.1094	30.49	69.51	0.0734	30.09	69.91
2006	0.1064	29.40	70.60	0.0719	27.74	72.26
2007	0.0795	29.82	70.18	0.0672	31.38	68.62
2008	0.0781	33.54	66.46	0.0616	30.93	69.07
2009	0.0732	30.86	69.14	0.0631	32.02	67.98
2010	0.0714	30.70	69.30	0.0660	28.04	71.96
2011	0.0697	30.78	69.22	0.0581	31.01	68.99
2012	0.0685	30.15	69.85	0.0594	31.85	68.15
2013	0.0671	30.43	69.57	0.0598	31.91	68.09
2014	0.0645	30.41	69.59	0.0581	29.50	70.50
2015	0.0593	31.58	68.42	0.0643	25.42	74.58
2016	0.0727	26.71	73.29	0.0675	20.93	79.07
2017	0.0825	23.10	76.90	0.0791	21.66	78.34
2018	0.0806	25.11	74.89	0.0849	19.85	80.15
2019	0.0908	22.18	77.82	0.0719	24.75	75.25
2020	0.0944	22.19	77.81	0.0809	21.68	78.32
2021	0.0978	23.27	76.73	0.0827	19.89	80.11

**Fig. 6:**
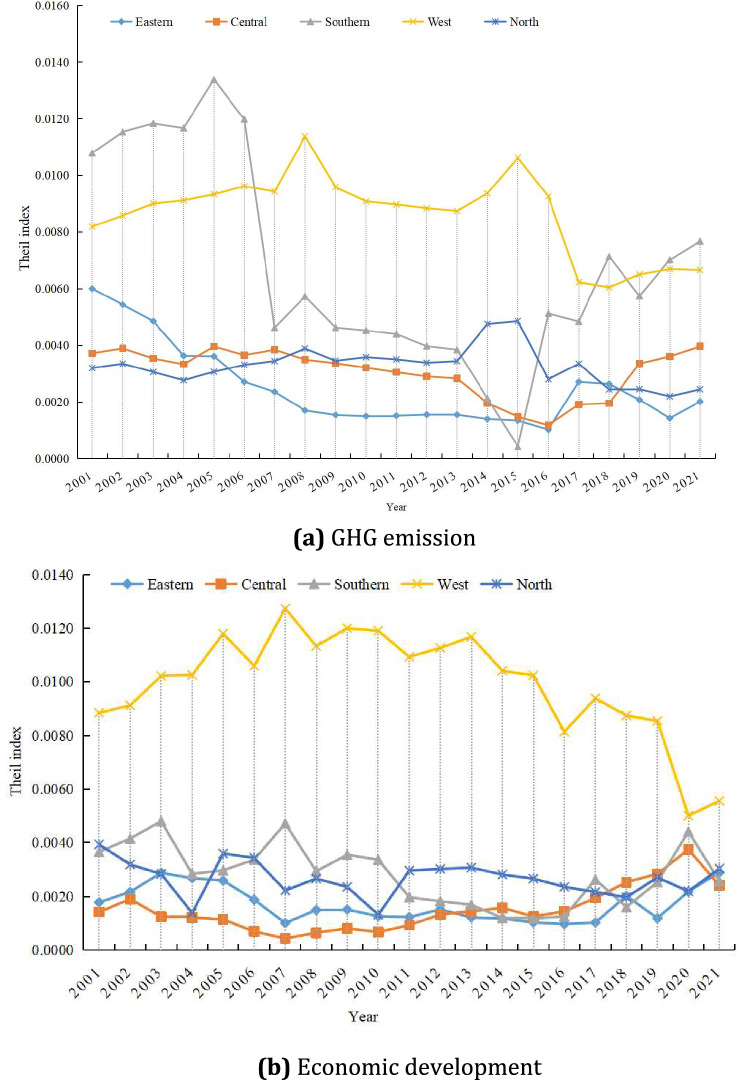
Changes in regional disparities between livestock GHG emissions and economic development by region in Henan Province, 2001–2021.

The regional variations in GHG emissions and livestock production’s economic development across Henan Province are depicted in Fig. 6. From the standpoint of the shifting trend in GHG emissions, the Theil index of GHG emissions in other regions exhibited a steady decline, except central Henan, where it increased marginally from 0.0037 to 0.0040. Among these, the southern Henan Theil index of GHG emissions rose from 0.0108 in 2001 to 0.0134 in 2005, while the western Henan Theil index of GHG emissions rose from 0.0082 in 2001 to 0.0106 in 2015. The fluctuation range of the Theil index in northern Henan is small, and the development is relatively stable. From the perspective of the trend of economic development, the Theil index of cities in western Henan decreased from 0.0088 to 0.0056, but compared with other regions, the gap in economic development level in the region is still at a high level. Overall, the regional gap in economic development in most regions has shown an upward trend year on year, although the central Henan region has slightly recovered after 2020, and the change is relatively stable^33^.

### Analysis of the decoupling effect of GHG emissions from livestock farming in Henan Province

Using the estimated GHG emissions from Henan Province’s livestock industry for 2001–2021 and historical data on the total value of livestock production in the region, the decoupling elasticity coefficient and decoupling status of Henan Province’s livestock industry GHG emissions can be obtained using formula (10) and Table 4. The results are shown in Table 7.

**Table 7:** Decoupling elasticity index for Henan Province, 2001 ~ 2021.

Year	∆CO_2_	∆GDP	Decoupling elasticity values(E)	Decoupling States
2001–2002	0.016	0.125	0.124	D
2002–2003	0.082	0.089	0.920	QI
2003–2004	0.089	0.327	0.271	D
2004–2005	0.060	0.124	0.488	D
2005–2006	0.067	− 0.151	− 0.440	GI
2006–2007	− 0.306	0.215	− 1.421	G
2007–2008	0.059	0.358	0.165	D
2008–2009	0.017	− 0.064	− 0.270	GI
2009–2010	− 0.009	0.049	− 0.186	G
2010–2011	− 0.007	0.202	− 0.037	G
2011–2012	0.010	0.015	0.679	D
2012–2013	0.018	0.087	0.207	D
2013–2014	0.022	− 0.003	− 8.452	GI
2014–2015	0.004	− 0.032	− 0.115	GI
2015–2016	− 0.027	0.053	− 0.502	G
2016–2017	− 0.233	0.005	− 43.058	G
2017–2018	− 0.010	− 0.067	0.154	DI
2018–2019	− 0.033	0.047	− 0.687	G
2019–2020	− 0.065	0.233	− 0.278	G
2020–2021	0.122	0.030	4.038	Q
2001–2002	0.016	0.125	0.124	D

Note: D denotes weak decoupling, DI denotes weak negative decoupling, G denotes strong decoupling, GI denotes strong negative decoupling, Q denotes expansive negative decoupling, N denotes recessive decoupling, QI denotes expansive coupling, and NI denotes recessive coupling.

Table 7 illustrates the tendency of weak decoupling to strong decoupling by separating the decoupling elasticity of GHG emissions and economic growth in the livestock farming sector of Henan Province into three states: weak decoupling, strong decoupling, and strong negative decoupling. The decoupling trend is consistent with GHG emissions, according to the time series. The weak decoupling condition dominated from 2001 to 2006, with GHG emissions growing in line with the economy and the GHG emissions curve on an upward trend, but the growth rate of GHG emissions was much lower than the GDP growth rate, suggesting that the livestock industry, while pursuing production capacity and output, also constrained GHG emissions to some extent^33^. The period 2005–2006 belongs to the strong negative decoupling state, which means that the GHG emissions of the livestock industry continued to increase while the output value of the livestock industry decreased, as the output value of the livestock industry decreased in 2006 due to the impact of animal diseases^33^. In the stage of large fluctuations, in which the rate of change of GHG emissions of the livestock industry and the growth rate of an economic output value of Henan Province are extremely unstable, and the economic output value of livestock in 2008–2009, 2013–2015 even negative growth, so that the decoupling relationship in this period appeared strong decoupling, weak decoupling, weak negative decoupling three kinds of state; from 2015 to 2021, the decoupling elasticity of livestock GHG emissions in Henan Province and the economic growth state is dominated by the strong decoupling state. This indicates that carbon emissions show negative growth while the overall economy continues to develop and grow^33^. This is because, in 2016, the General Office of the People’s Government of Henan Province issued the "Work Arrangements for Energy Conservation, Emission Reduction and Carbon Reduction in Henan Province in 2016", which came into effect. This indicates that the energy conservation and emission reduction measures have achieved good results, but further efforts are still needed to achieve a more comprehensive and stronger decoupling. However, an expansionary negative decoupling was observed in 2020–2021, which may be related to the policy easing and increased energy demand during the economic recovery process after the epidemic, leading to a significant increase in emissions. Overall, the livestock sector in Henan Province is moving towards a balance between economic growth and GHG emission control, but the risk of a rebound in GHG emissions remains a concern^33^.

### Dynamic evolution characteristics of GHG emissions from livestock farming in Henan Province

Using the traditional Markov chain model and the spatial Markov chain model, the dynamic evolution law of GHG emissions from livestock farming in Henan Province was studied. Among them, I, II, III, and IV represent the four types of low level, medium–low level, medium–high level, and high level, respectively. The state transfer probability of GHG emissions from the livestock industry in Henan Province was measured using the traditional Markov chain, and the results are shown in Table 8^33^. The value on the diagonal is the possibility that a city remains in the same state in the next period, that is, the self-locking probability. For Henan Province, the values represented on the diagonal line are more than those represented on the non-diagonal line. The maximum value observed on the diagonal line is 89.47%, while the minimum value is 67.12%. It shows that the probability of GHG emission types of livestock farming in each city remaining unchanged is at least 67.12%, which is significantly higher than the probability of transfer of GHG emission types, indicating that the change of GHG emission of regional livestock farming is constrained by the initial GHG emission types and stocks, exhibiting growth inertia and path dependence. The values on the non-diagonal line are not all zero, and the majority of the non-zero values are situated in the ‘adjacent area’ of the diagonal line. This indicates that there is a possibility of upward or downward transfer of GHG emissions in the region, but there is no transition (cross-level transfer of GHG emission types). The reason may be that the development of livestock farming is continuous, and it is unlikely to have a cross-level transition of GHG emission types in the absence of significant national strategy implementation and industrial development opportunities^33^.

**Table 8:** Conventional Markov transfer probability matrix for GHG emissions from livestock in Henan province.

t/t + 1	I	II	III	IV
I	0.8947	0.1053	0.0000	0.0000
II	0.2877	0.6712	0.0411	0.0000
III	0.0375	0.2000	0.6750	0.0875
IV	0.0000	0.0128	0.1923	0.7949

At the same time, this paper employs a Markov transition probability calculation based on the spatial weight matrix to further examine the influence of GHG emissions from livestock farming in neighbouring cities on the city (Table 9). It can be seen that the self-locking probability of Type I cities is 0,93.75%, 97.22%, and 25%, respectively, 100%, 56.25%, 70%, and 90% for Type II cities, 0, 72%, 66.67%, and 57.14% for Type III cities, and 0, 0, 85.45%, and 65.22% for Type III cities^33^. Therefore, (1) Livestock GHG emissions in Henan Province are greatly affected by urban spatial lag, and the self-locking probability has changed significantly compared with the results of the conventional Markov chain matrix. Overall, in the spatial Markov transfer matrix, low-level cities are more vulnerable to the impact of neighbouring cities, and the higher the level of neighbouring cities, the lower the self-locking probability of the city, and the greater the transfer probability^33^. (2) The growth of GHG emissions from livestock farming in adjacent cities will affect the city, and the cities with medium–high and high spatial lags have the most significant impact on the growth of GHG emissions in their city. In the case of considering neighbourhood lag types, the probabilities of transition from a low to a high level are 0, 6.25%, 3.33%, and 0, respectively, and the probabilities of transition from medium–high level to a high level are 0, 0, 8.33%, and 42.86% respectively, which indicates that the higher the GHG emission of livestock farming in adjacent cities, the more likely it is to increase the GHG emission of livestock farming in this city. However, it should be noted that for some cities with low GHG emissions, there may be a risk of reducing the positive spillover effect if the gap between neighbouring cities and their development is too large. In summary, the GHG emissions from livestock production in Henan Province are restricted by geographical factors, and the level of GHG emissions in one city will have a differentiated impact on its neighbouring cities. From the overall trend, when the spatial lag type of a city’s adjacent cities is medium–high and high-level, the probability of that city achieving a state jump increases; while when it is adjacent to low and medium–low level cities, the probability of upward transfer decreases, and the probability of downward transfer increases.

**Table 9:** Spatial transfer probability matrix of GHG emissions from livestock farming in Henan Province.

Adjacent type	t/t + 1	I	II	III	IV
I	I	0.0000	1.0000	0.0000	0.0000
	II	0.0000	1.0000	0.0000	0.0000
	III	0.0000	0.0000	0.0000	0.0000
	IV	0.0000	0.0000	0.0000	0.0000
II	I	0.9375	0.0625	0.0000	0.0000
	II	0.3750	0.5625	0.0625	0.0000
	III	0.0000	0.2800	0.7200	0.0000
	IV	0.0000	0.0000	0.0000	0.0000
III	I	0.9722	0.0278	0.0000	0.0000
	II	0.2667	0.7000	0.0333	0.0000
	III	0.0625	0.1875	0.6667	0.0833
	IV	0.0000	0.0000	0.1455	0.8545
IV	I	0.2500	0.7500	0.0000	0.0000
	II	0.1000	0.9000	0.0000	0.0000
	III	0.0000	0.0000	0.5714	0.4286
	IV	0.0000	0.0435	0.3043	0.6522

## Discussion

As the ‘dual carbon’ goals are being implemented, the green and low-carbon development of the livestock industry plays a crucial role in reducing GHG emissions. Currently, the primary methods for estimating carbon emissions include LCA, I-O, and the IPCC factor method. The IPCC factor method primarily considers carbon emissions during the production process of livestock and poultry farming; China’s input–output tables are updated every 5 years, and there are significant differences in the carbon emission intensity per unit of output reflected in the statistical data of the livestock industry across regions, leading to time lag and variability when using this method to estimate carbon emissions; LCA is a ‘bottom-up’ process-based analysis method that considers all production and usage stages of the research object, resulting in high reliability. This study takes Henan Province, a major livestock-producing province, as the research object, conducts research at the prefecture-level city administrative unit level, and uses the LCA method to estimate GHG emissions by stage and region.

This study found that GHG emissions from the livestock industry in Henan Province showed a fluctuating downward trend from 2001 to 2021 (with an average annual decrease of 1.18%), consistent with the overall trend of GHG emissions from the livestock industry at the national level^23^, thereby validating the impact of carbon reduction policies at the provincial level. Although GHG emissions decreased significantly during the two periods of 2001–2007 and 2015–2021, which generally aligns with the GHG emission trends reported by Xie et al.^66^ and Chen et al.^67^ for Henan Province, the underlying mechanisms differ: The decline in emissions from 2001 to 2007 was caused by short-term shocks such as porcine reproductive and respiratory syndrome (PRRS), combined with factors like low feed utilisation rates and significant fluctuations in livestock scale under extensive farming practices^62^, while the reduction from 2015 to 2021 was achieved through industrial restructuring and measures such as reducing cattle inventory, which aligns with the implementation of national low-carbon livestock policies (e.g., promoting large-scale farming and resource utilization of manure)^68^. The emission reduction effects during this period are consistent with the national trend of ‘policy-driven emission reduction’^23^. In recent years, Henan Province has vigorously promoted the resource utilisation of livestock manure and developed a circular agriculture system combining crop cultivation and animal husbandry. The province has achieved significant results in the green and circular development of its livestock industry^69^. By linking total emissions to feed grain production, energy consumption, transportation, and processing, the importance of indirect emissions has been confirmed, providing quantitative evidence for emissions reduction across the entire supply chain. This indicates that Henan Province has shifted from emissions reduction in single stages to systematic governance. However, compared to provinces with higher levels of livestock modernisation (such as Jiangsu)^70^, there is still a gap in the control of indirect emissions. This approach is more systematic than research that only focuses on the farming stage^71^.

At the same time, this study found that during the study period, the spatial and dynamic evolution characteristics of GHG emissions from the livestock industry in Henan Province both showed regional development imbalances. Specifically, the GHG emission Theil indices across regions showed a declining-then-increasing trend during the study period. The eastern Henan region has developed a complete industrial chain encompassing ‘feed cultivation-large-scale farming-food processing’; however, the western Henan region still primarily relies on traditional small-scale farming as its main production model. This disparity in industrial structure resulted in regional disparity contribution rates ranging from 66 to 77%, though these rates generally showed an upward trend year by year. Based on the Tapio model, the decoupling relationship between GHG emissions and output value in Henan Province’s livestock industry was analysed. The strong decoupling state after 2015 aligns with the ‘Henan Province 2016 Energy Conservation, Emission Reduction, and Carbon Reduction Work Plan,’ consistent with national policy decisions aiming to achieve both carbon reduction and economic growth through policy intervention^69^. While the expansive negative decoupling observed in 2020–2021 aligns with the post-pandemic emissions rebound nationwide, demonstrating that the stability of decoupling states can be disrupted by sudden events. In terms of dynamic evolution distribution, livestock GHG emissions are constrained by geographical factors and industrial structure. The GHG emission levels of a city itself can have differentiated impacts on its neighbouring cities. Therefore, to better implement the ‘dual carbon’ development goals for the livestock sector, it is urgent to implement regional, graded, and categorised emission reduction strategies for the livestock farming industry.

**Fig. 7:**
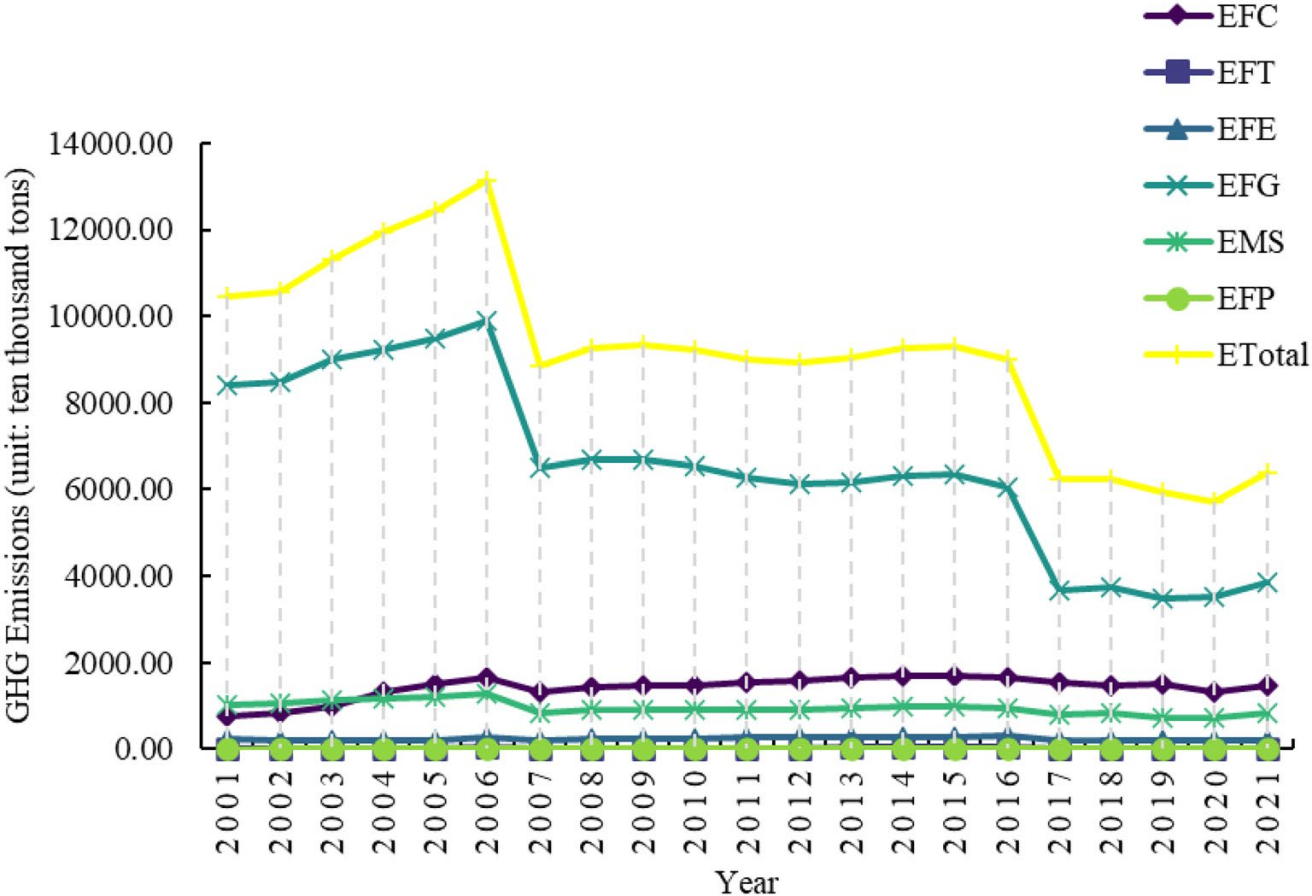
GHG emissions from livestock farming and various segments in Henan Province (The GWP20 values of methane and nitrous oxide are taken from the values in Table 7.SM.7 in Forster et al.^72^, which are 81.2 and 273, respectively).

To enhance the comprehensiveness of the research, we supplemented the estimates of GWP20 based on Fig. 3 and Table 5 as shown in Fig. 7 and Table 10, and compared them with the results based on GWP100 to analyze their impact on the robustness of conclusions. GWP100 is a widely used standard index in international climate policy, such as in the Paris Agreement and the Kyoto Protocol, focusing on the long-term impacts of climate change. In contrast, GWP20 emphasizes the warming potential of short-lived greenhouse gases (SLGHG) like methane, paying more attention to short-term climate impacts. A comparison of the results under the two indices reveals that:

**Table 10:** Rates of change of GHG emissions and their components from livestock farming in Henan Province, 2001–2007 and 2015–2021. (The GWP20 values of methane and nitrous oxide are taken from the values in Table 7. SM.7 in Forster et al.^72^, which are 81.2 and 273, respectively).

Emissions from different sectors	2001–2007 Year	2015–2021 Year
ETotal	− 2.68%	− 6.10%
EFC	9.08%	− 2.41%
EFT	9.50%	− 2.82%
EFE	− 3.19%	− 4.42%
EFG	− 4.21%	− 7.96%
EMS	− 2.79%	− 2.49%
EFP	1.83%	− 0.27%

1. During the two research periods (2001–2007 and 2015–2021), the ETotal from livestock in Henan Province showed a downward trend, regardless of whether GWP100 or GWP20 was used, indicating that the conclusion regarding emission reduction is robust. However, the reduction magnitude estimated under GWP20 (− 2.68% and − 6.10%) was greater than that under GWP100 (− 1.05% and − 4.67%). This difference is primarily attributed to the short-term impacts of factors such as porcine reproductive and respiratory syndrome and avian influenza on CH_4_ emissions during the 2001–2007 period under GWP20. In the period from 2015 to 2021, the gap between GWP20 and GWP100 further widened, with a decline in ETotal under GWP20 (6.10%) being 1.43 percentage points higher than that under GWP100 (4.67%), mainly due to the sharp reduction in cattle stock and the synergistic effects of policies promoting the utilization of livestock manure for carbon reduction.
  As shown in Fig. 7, emissions in 2007 decreased by approximately 32.57% compared to 2006, while the reduction shown in Fig. 3 is about 30.59%. This is due to a decline in livestock production capacity caused by the pandemic over 1–2 years, a typical 'short-term shock’. The sensitivity of SLGHG to the time scale of GWP20 captures this characteristic, whereas the 100-year average effect of GWP100 smooths out short-term fluctuations, leading to an underestimation of the peak. Compared to GWP100, the ‘decline slope’ of emissions from 2015 to 2021 is steeper. This difference indicates that policies such as strengthening the integration of planting and breeding and promoting the utilization of livestock manure resources in Henan Province’s 14th Five-Year ecological planning are more significant in the short term than in the long term. If only GWP100 is used, the contribution of these policies to 'medium and long-term climate goals’ (such as the carbon peak by 2030) would be underestimated.
2. When using GWP20, the share of CH_4_ emission sources (mainly EFG) in total emissions will significantly increase. This may lead decision-makers to focus more on reducing climate impacts by improving feed formulations when developing short-term emission reduction strategies. However, this study also indicates that segments primarily composed of CO_2_ emissions, such as feed crop planting (EFC) and feed crop transportation and processing (EFT), show consistent results in their rate of change under both GWP values, indicating that the assessment of these segments is not affected by the choice of time scale.
3. Although the absolute values differ, these two indicators suggest that the adjustment of industrial structure (such as reducing the number of cattle) and policy guidance are important factors in promoting the reduction of GHG emissions in the livestock sector of Henan Province. Therefore, the conclusions drawn from this study regarding emission trends, dominant reduction sectors, and policy effectiveness are robust. Overall, the choice of GWP values is based on value judgments concerning the time frame of policy goals (short-term risk warning and long-term temperature control). This study also provides two results aimed at increasing transparency and providing policymakers with more comprehensive information. Interpreting the corresponding results according to different climate governance goals (short-term mitigation or long-term stabilization) is recommended.

Due to data collection limitations, this paper still has some shortcomings. Data from 2021 onwards may not be publicly available due to statistical cycle reasons, so it is impossible to obtain complete and reliable raw data for research purposes. Therefore, the analysis is based solely on available data from before 2021. In the future, if more detailed data becomes publicly available, conducting a more refined analysis of GHG emissions and their influencing factors will be possible, thereby proposing more systematic emission reduction strategies. Additionally, combining deep learning with the prediction and analysis of GHG emissions from the livestock industry is a direction worthy of further exploration in the future.

## Conclusions and policy recommendations

### Conclusions

This study estimates GHG emissions from the livestock industry in Henan Province, reveals their temporal and spatial variations and regional characteristics, and conducts a spatial analysis of GHG emissions from the livestock industry. This is not only of great significance for promoting the high-quality development of the livestock industry in Henan Province, but also fills the gap in GHG emissions research on Henan Province, a major livestock-producing province, and provides a reference for other regions to achieve GHG emission reductions and livestock industry development. The conclusions of this study are as follows:

1. From 2001 to 2021, GHG emissions from the livestock industry in Henan Province showed an overall fluctuating downward trend, with two significant decreases occurring between 2001 and 2007 and between 2015 and 2021. The first decline was primarily influenced by factors such as depressed pig prices, rising feed costs, and epidemics. The second decline was achieved through optimising the structure of cattle herds, significantly reducing GHG emissions. Additionally, this was closely tied to policy guidance promoting green and low-carbon development in the livestock sector since the launch of ecological civilisation initiatives. From a regional perspective, Henan Province’s livestock sector has gradually formed a large GHG emission zone extending from the northwest to the southeast, with a distinct ‘higher in the east, lower in the west’ regional disparity.
2. The Henan Province livestock industry GHG emissions Theil index has shown a consistent downward trend, decreasing from 0.1064 in 2006 to 0.0978 in 2021, with significant regional differences that are gradually narrowing. In contrast, the economic development Theil index exhibits a ‘first decline, then rise’ trend, indicating that the overall gap in economic development between cities is widening.
3. The decoupling elasticity between GHG emissions from livestock farming and economic growth in Henan Province is in three states: weak decoupling, strong decoupling, and strong negative decoupling, showing a trend from weak decoupling to strong decoupling. This indicates that environmental protection and economic development have achieved an overall positive interaction, with improved resource utilisation efficiency and effective environmental governance. However, there are significant differences in the decoupling states between different regions, and the relationship between economic growth and GHG emissions remains unstable, with many regions in a state of weak decoupling. Additionally, the number of regions exhibiting strong negative decoupling and expanding negative decoupling continues to increase. These trends indicate that regional ecological environments and economic development have not yet been fully coordinated.
4. The results of the traditional Markov transition probability matrix analysis show that the self-locking probability (the likelihood of maintaining the current state) for each city’s livestock GHG emission type (low, medium–low, medium–high, high) is significantly higher than the transition probability, with a minimum value of 67.12% and a maximum value of 89.47%. The spatial Markov transition probability matrix incorporating the influence of neighbouring cities shows that when a city’s neighbouring cities belong to the medium–high or high emission categories, the former’s likelihood of achieving a state transition increases. Conversely, if neighbouring cities are low or medium–low emission categories, the likelihood of upward movement decreases, while the likelihood of downward movement increases.

### Policy proposal

1. Optimising feed nutrient composition to promote integrated livestock and crop production. Given that the decline in cattle inventory has made a significant contribution to emissions reduction, policies supporting large-scale livestock farming should continue to be implemented. Additionally, adjusting feed composition to control daily intake is the most direct method to reduce methane emissions from gastrointestinal fermentation^73,74^, thereby lowering GHG emissions per unit of product. Depending on the method or nature of nutritional intervention, this approach can reduce methane emissions from ruminant animals by up to 70%^75^. In addition, the coordinated development of planting and breeding should be encouraged, and the integrated management of agriculture and animal husbandry should be carried out to form a circular mode of 'breeding-planting-breeding’. For example, by making full use of livestock and poultry excrement, collecting manure and processing it into organic fertiliser for planting, and using the straw produced by planting as feed for livestock and poultry breeding, the resource circulation and transformation between agriculture and livestock farming can be realised, and GHG emissions can be further reduced.
2. Implement differentiated management to promote low-carbon livestock farming. GHG emissions from the livestock industry in Henan Province vary significantly across regions. It is recommended that the government adopt appropriate preferential policies for major livestock-producing cities (such as the southern Henan region) and establish a compensation system for GHG emissions, providing differentiated compensation. For low-carbon emission regions (such as western Henan), their existing low-carbon advantages should be fully leveraged to further optimise the allocation of livestock production resources, gradually reduce the proportion of traditional livestock farming, and actively develop emerging industries. Additionally, further efforts should be made to encourage low-carbon pilot cities to achieve an early decoupling of carbon emissions from economic development, thereby promoting inter-regional industrial cooperation and technology transfer, and establishing a framework where economically developed regions lead Henan Province’s low-carbon development.
3. Strengthen government environmental regulations and improve the GHG emissions reduction supervision system. Based on the development level of the livestock industry and regional differences, establish a scientific and reasonable regional GHG emissions reduction plan for livestock production with the specific goal of reducing GHG emissions from livestock production. Strengthen comprehensive management of CH₄ and N₂O emissions from livestock farming to address GHG emissions issues in livestock production. To ensure the effective implementation of environmental regulations, it is essential to establish and improve the GHG emissions supervision system. Relevant laws and regulations should be formulated to clarify the standards and reduction targets for GHG emissions in the livestock industry, and self-supervision should be strengthened to institutionalise the content and methods of GHG emissions supervision. This will enable decision-makers and management departments in Henan Province to promptly and effectively obtain GHG emissions data from various cities and make informed decisions.

## Data Availability

The data used to support the findings of this study are available from the corresponding author upon reasonable request.
